# Genetic risk factors for variant Creutzfeldt–Jakob disease: a genome-wide association study

**DOI:** 10.1016/S1474-4422(08)70265-5

**Published:** 2009

**Authors:** Simon Mead, Mark Poulter, James Uphill, John Beck, Jerome Whitfield, Thomas EF Webb, Tracy Campbell, Gary Adamson, Pelagia Deriziotis, Sarah J Tabrizi, Holger Hummerich, Claudio Verzilli, Michael P Alpers, John C Whittaker, John Collinge

**Affiliations:** a Medical Research Council Prion Unit and Department of Neurodegenerative Disease, Institute of Neurology, Queen Square, London, UK; b Papua New Guinea Institute of Medical Research, Goroka, East Highlands Province, Papua New Guinea; c Centre for International Health, Curtin University, Perth, Australia; d Department of Epidemiology and Population Health, London School of Hygiene and Tropical Medicine, London UK

## Abstract

**Background:**

Human and animal prion diseases are under genetic control, but apart from *PRNP* (the gene that encodes the prion protein), we understand little about human susceptibility to bovine spongiform encephalopathy (BSE) prions, the causal agent of variant Creutzfeldt–Jakob disease (vCJD).

**Methods:**

We did a genome-wide association study of the risk of vCJD and tested for replication of our findings in samples from many categories of human prion disease (929 samples) and control samples from the UK and Papua New Guinea (4254 samples), including controls in the UK who were genotyped by the Wellcome Trust Case Control Consortium. We also did follow-up analyses of the genetic control of the clinical phenotype of prion disease and analysed candidate gene expression in a mouse cellular model of prion infection.

**Findings:**

The *PRNP* locus was strongly associated with risk across several markers and all categories of prion disease (best single SNP [single nucleotide polymorphism] association in vCJD p=2·5×10^−17^; best haplotypic association in vCJD p=1×10^−24^). Although the main contribution to disease risk was conferred by *PRNP* polymorphic codon 129, another nearby SNP conferred increased risk of vCJD. In addition to *PRNP*, one technically validated SNP association upstream of *RARB* (the gene that encodes retinoic acid receptor beta) had nominal genome-wide significance (p=1·9×10^−7^). A similar association was found in a small sample of patients with iatrogenic CJD (p=0·030) but not in patients with sporadic CJD (sCJD) or kuru. In cultured cells, retinoic acid regulates the expression of the prion protein. We found an association with acquired prion disease, including vCJD (p=5·6×10^−5^), kuru incubation time (p=0·017), and resistance to kuru (p=2·5×10^−4^), in a region upstream of *STMN2* (the gene that encodes SCG10). The risk genotype was not associated with sCJD but conferred an earlier age of onset. Furthermore, expression of *Stmn2* was reduced 30-fold post-infection in a mouse cellular model of prion disease.

**Interpretation:**

The polymorphic codon 129 of *PRNP* was the main genetic risk factor for vCJD; however, additional candidate loci have been identified, which justifies functional analyses of these biological pathways in prion disease.

**Funding:**

The UK Medical Research Council.

## Introduction

Prion diseases are transmissible, fatal, neurodegenerative conditions of human beings and animals that are caused by the autocatalytic misfolding of host-encoded prion protein (PrP).^1^ An epizootic prion disease, bovine spongiform encephalopathy (BSE), widely exposed the population of the UK (and, to a lesser extent, many other populations) to prion infection. The subsequent diagnosis of variant Creutzfeldt–Jakob disease (vCJD) in young British adults, and the experimental finding that this was caused by BSE-like prions,^2^, ^3^, ^4^ resulted in a major public and animal health crisis.

Although the number of recorded clinical cases of vCJD to date has been small (∼200) in relation to the millions of people who were potentially exposed, how many individuals were infected is unclear. The clinically silent incubation period in human beings can exceed 50 years,^5^ and estimates of the prevalence of subclinical infection made on the basis of screening archived surgical specimens predicts that thousands of individuals in the UK are infected.^6^ Blood transfusion seems to be an efficient route of secondary transmission^7^ but no screening test to ensure the safety of blood products is yet available. Case control studies have identified no unusual occupational, dietary, or other exposure to BSE prions among patients with vCJD,^8^ which suggests that genetic factors might be crucial.

A known genetic factor for susceptibility to prion disease is the common single nucleotide polymorphism (SNP) at codon 129 in *PRNP*, the gene that encodes PrP in human beings. Here, either methionine (∼60% allele frequency in Europeans) or valine is encoded.^9^ All patients with vCJD who have been genotyped are homozygous for methionine,^10^ which represents the strongest association to date of a common genotype with any disease. Although this is a powerful effect, about a third of the exposed UK population have this genotype. An important role for other genetic loci is supported by the results of mouse quantitative trait locus studies, which have identified many regions that are not linked to *Prnp* but control the highly variable prion disease incubation periods,^11^, ^12^ including that of BSE prions.^13^ The importance to public health of understanding susceptibility to BSE prion infection in human beings is therefore clear.

We undertook a genome-wide association study with 100K and 500K Affymetrix arrays with all available samples from white British patients with vCJD (n=119) compared with our own and publicly available UK control data, which was genotyped by the Wellcome Trust Case–Control Consortium (WTCCC). Because all available vCJD samples from the UK were included in the discovery phase, we went on to compare the top-ranked SNP associations and additional SNPs at the *PRNP* locus with a large and diverse collection of patients with prion disease, including those with iatrogenic CJD (iCJD), sporadic CJD (sCJD), and kuru.

## Methods

### Samples

**Figure 1:**
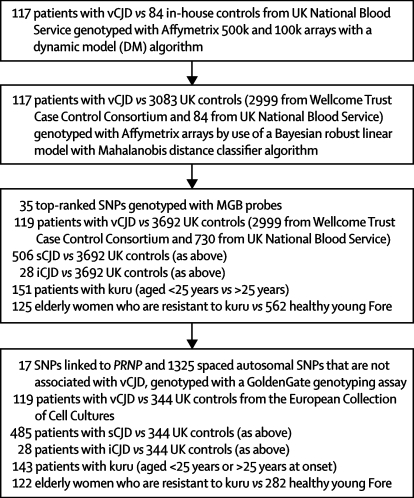
Flowchart of the genotyping in the tiered study For each tier, the patient and control sample collections used are subsets of those genotypes in the minor groove-binding (MGB) probe study. In the first two tiers, the 117 samples from patients with vCJD are a subset of the 119 used in tiers three and four. In the second tier, the 84 samples from the UK National Blood Service are a subset of the 730 used in the third tier. In the final tier, the 485 samples from patients with sCJD, the 143 samples from patients with kuru, the 122 samples from elderly women, and the 282 samples from healthy young Fore are all subsets of the samples used in the third tier.

Figure 1 shows the four tiers of genotyping in the study. Samples were obtained from 119 patients with vCJD (ten patients with probable vCJD and 109 patients with definite vCJD) who were diagnosed at the National Prion Clinic (NPC), London, or the National CJD Surveillance Unit (NCJDSU), Edinburgh, between 1995 and 2005 according to established criteria. Patients who acquired iatrogenic vCJD through blood transfusion were not included in this series. All patients with vCJD were thought to have acquired the disease in the UK and were of white British ethnic origin (60% were men; mean age of disease onset was 29·8 [SD 10·9] years).

Samples were obtained from 506 patients with probable or definite sCJD diagnosed according to established criteria and from 28 patients with iCJD related to exposure to cadaver-derived growth hormone in the 1980s or earlier; these samples were obtained from the NPC or the NCJDSU or from other clinical colleagues in the UK. All patients were from the UK or elsewhere in northern Europe. Although most patients were of white British ethnic origin, and all patients of known non-white ethnic origin were excluded, this information was based on names and geographical location for some samples. 325 patients had pathologically confirmed sCJD and 181 patients had a diagnosis of probable sCJD with a high specificity according to published WHO criteria, although some of these patients might have had a neuropathological diagnosis made elsewhere.^14^ Mean age of disease onset was 68·2 (SD 12·0) years for the patients with sCJD and 31·1 (6·3) years for the patients with iCJD. 50% of the samples from patients with sCJD were from men.

Before 1987, kuru surveillance was done by many different investigators; however, from 1987 to 1995 surveillance was done solely by the Kuru Surveillance Team of the Papua New Guinea Institute of Medical Research. From 1996, kuru surveillance was strengthened: a field base and basic laboratory for sample processing and storage were established in the village of Waisa in the South Fore, and a wide collection of population control samples were taken.^5^ The samples from patients with kuru (n=151) were taken from young children, adolescents, and adults during the peak of the epidemic and from recent cases of kuru with long incubation times in elderly patients. The patients lived in the South Fore (n=53), North Fore (n=40), Gimi (n=3), and Keiagana (n=10) regions; linguistic group was not known in 45 patients.

Elderly women who had been exposed to kuru were defined as aged older than 50 years in 2000 and from a region that had been exposed to kuru: South Fore (n=74), North Fore (n=36), Gimi (n=13), and Keiagana (n=2). The modern-day healthy population from the exposed region was obtained by matching each elderly woman to at least two current residents of the same village who were aged less than 50 years in 2000. These mostly came from the South Fore, with some from the North Fore, and a small number of individuals from Gimi, Keiagana, and Yagaria linguistic groups, as indicated. First-degree relatives of the elderly women, identified by either genealogical data or microsatellite analysis, were excluded from these groups.

155 samples were from volunteers recruited by the Medical Research Council Prion Unit from the National Blood Service (NBS). Information was collected about their sex, age, ethnic origin, and birthplace divided into 12 regions. 90 samples genotyped with Affymetrix arrays were selected to match the vCJD collection for white British ethnic origin, birthplace (by 12 regions in UK, each region was represented in patients and controls with the same ranking), and sex (proportion of men with vCJD was 60%, and the proportion of men in the NBS controls was 57%).

A further 575 UK control samples were obtained for the replication phases of the study (730 healthy controls in total) from the NBS (95 white, random, healthy young blood donors) and from the European Collection of Cell Cultures (ECACC) human random control DNA collection (480 blood donors of known age and sex). No selection was done in the replication phase of the study. Not all control samples were genotyped for all replication studies; however, there is no reason to expect significant genetic heterogeneity in our collections of UK blood donors based on analyses of the UK population done by the WTCCC and others.^15^ All UK control samples contained good quality unamplified DNA. The mean age at sampling was 38·7 (SD 10·8) years, and 51% were men. In addition, we used publicly available UK control data generated by the WTCCC. In brief, 1500 samples from the 1958 British Birth Cohort and 1500 samples from the UK Blood Service Control Group were genotyped with commercial Affymetrix 500K arrays with a Bayesian robust linear model with Mahalanobis distance (BRLMM) algorithm. We did not detect any duplicate individuals between the UK control collections nor any significant differences in allele frequency between our in-house UK control collections or those genotyped by the WTCCC.

The clinical and laboratory studies were approved by the local research ethics committee of University College London Institute of Neurology and National Hospital for Neurology and Neurosurgery and by the Medical Research Advisory Committee of the Government of Papua New Guinea. The full participation of the Papua New Guinea communities was established and maintained through discussions with village leaders, communities, families, and individuals. Most of the UK samples were obtained with written consent from patients or next of kin; however, where this was not available, for example, for archival vCJD tissue obtained at post-mortem examination, we obtained the specific approval of our local ethics committee for the use of these samples in the research.

### Procedures

For the samples from patients with vCJD, genomic DNA was mostly extracted from peripheral blood, although 45 samples were extracted from brain tissue. For a few samples, whole-genome amplification, either with a φ29 protocol called multiple displacement amplification (MDA; Geneservice, Cambridge, UK; ten samples) or GenomePlex Complete Whole Genome Amplification Kit (WGA2; Sigma, UK; two samples) was necessary.

For the samples from patients with sCJD or iCJD, whole-genome amplification with either MDA in 138 samples or WGA2 in 29 samples was needed. Most of the samples from patients with sCJD and iCJD were extracted from blood, although DNA from eight samples in the iCJD group and 70 samples from the sCJD group was derived from brain tissue. 112 samples from patients with sCJD were sent as DNA to the MRC Prion Unit for analysis, most of which were extracted from blood. Genomic DNA was usually extracted from peripheral blood. PAXgene blood-derived RNA samples were also collected (Reanalytix, QIAGEN, UK).

DNA from degraded archival kuru sera was isolated by QIAamp Blood DNA minikit (QIAGEN, UK) followed by whole-genome amplification with WGA2 in all but seven samples. The validation of this process for the degraded kuru samples has been reported elsewhere.^16^

Good-quality genomic DNA extracted from blood was available for 278 of 285 (98%) healthy controls from Papua New Guinea and 122 of 125 (98%) healthy elderly women with many exposures to kuru at mortuary feasts. All control samples from Papua New Guinea were extracted from blood.

All DNA samples were checked for degradation on 1% agarose gel and stored at 50 ng/μL in low-concentration tris-EDTA buffer.

Rocky Mountain Laboratory (RML) prion-infected mouse brain homogenate (0·001%) or mock-infected brain homogenate from wild-type CD-1 mice (0·001%) was used to infect GT-1 hypothalamic neuronal cells. 5000 cells were seeded into 96-well plates and incubated with either homogenate in standard growth medium (Opti-MEM supplemented with 10% fetal calf serum and 1% penicillin/streptomycin [Invitrogen, CA, USA]). The inoculum was removed after 3 days and the cells were split 1:8. Cells were then split 1:8 twice more at intervals of 3 days. High levels of prion infectivity were confirmed with the scrapie cell assay.^17^ Prion-infected and mock-infected cells were maintained in standard growth medium at 37°C in 5% CO_2_. Total RNA was extracted in triplicate with the RNeasy Midi kits (QIAGEN, UK) according to the manufacturer's instructions, from prion-infected and mock-infected cells that had been grown on 10-cm-diameter plates. RNA was eluted in RNAase-free water and stored at −80°C. RNA samples adjusted to a concentration of 250 ng in 5 μL were incubated at 50°C for 30 mins with an equal volume of Glyoxyl (Ambion, Warrington, UK) loading dye containing ethidium bromide. Samples were run on a 1·5% agarose mini-gel in 1× NorthernMAx glyoxyl-based gel prep and running buffer (Ambion) at 100 mV for 90 min to check sample integrity. RNA was then sent to AROS Applied Biotechnology AS (Denmark) for the following microarray analyses according to Affymetrix standard protocols: first and second strand complementary DNA synthesis was done with the SuperScriptII System (Invitrogen) from 5 μg RNA (a minor modification was made to the protocol by using an oligo-dT primer that contained a T7 RNA polymerase promoter site); labelled antisense RNA (cRNA) was prepared with the BioArray High Yield RNA Transcript Labelling Kit with biotin-labelled CTP and UTP (Enzo Life Sciences, NY, USA) and unlabelled NTPs. Unincorporated nucleotides were removed using RNeasy columns (QIAGEN). 15 μg of cRNA was fragmented, loaded on to the Affymetrix mouse expression array 430_2.0 probe array cartridge, and hybridised for 16 h. Arrays were washed, stained in the Affymetrix fluidics station and scanned with a confocal laser-scanning microscope (GeneChip Scanner 3000 System with Workstation and Autoloader).

The following sample comparisons were made in the association studies: vCJD versus UK controls genome-wide with Affymetrix array data; vCJD, sCJD, and iCJD versus UK controls in a validation and replication study with minor groove-binding [MGB] probes; healthy elderly women who were exposed to kuru at mortuary feasts versus geographically matched young individuals from the Eastern Highlands of Papua New Guinea in the replication study; young patients with kuru versus older kuru patients in the replication study. Figure 1 shows the tiered nature of the study. Subsets of each sample group have been used in previous studies of *PRNP* codon 129.^18^ The comparison of young versus old patients with kuru was based on a hypothesis derived from mouse models that states that genetic factors control the incubation time of human prion diseases.^12^ The incubation time of middle-aged or elderly patients who died of kuru at the peak of the epidemic cannot be calculated with precision and might have been many decades. Incubation times of up to 50 years or longer have been recorded in recently diagnosed patients,^5^ whereas children, adolescents, or young adults have a limited incubation time.^5^ Because the kuru collection was a mixture of samples from young and old patients, we hypothesised a priori that greater differences would be found between young people with kuru and old people with kuru than between people with kuru versus modern young healthy Fore. This strategy was supported by the precedent of homozygosity at codon 129 of *PRNP*, which was strongly associated with young versus old kuru, but was not significant in a comparison of all kuru with healthy Fore.

### Genotyping and statistical analysis

We used the Affymetrix 100K and 500K arrays (early access, EA-500K), which use four restriction enzymes in total. Our first case–control study used data generated by the Affymetrix DM (dynamic model) algorithm from 117 samples of patients with vCJD (two samples were not suitable for use with Affymetrix arrays) and 90 UK controls matched for birthplace. The 500K product is comprised of two arrays each of about 250K digested with the restriction endonucleases *Nsp*I or *Sty*I; the 100K product is comprised of two arrays each of about 50K digested with the restriction endonucleases *Xba*I and *Hind*I. Genotypes were called by the dynamic model (DM) and subsequently by BRLMM algorithms. Samples from patients with vCJD were repeated if the DM call rate was less than 85% or the BRLMM call rate was less than 90% and samples were excluded if they underperformed by these criteria (vCJD [n=0], NBS [n=6], WTCCC [n=5]). The median and mean BRLMM call rates (all non-WTCCC samples and all arrays) were 99·0% and 98·5%. No samples were excluded for excess or low heterozygosity. One duplicate sample but no related individuals were identified. With genome-wide SNP data, the PLINK toolset for whole-genome association and population-based linkage analysis enables estimates of the relatedness of individuals. For the purposes of confirming unrelatedness, this can be expressed as a probability for identity by descent (IBD)=0 using complete linkage agglomerative clustering. This probability was greater than 0·75 for all study pairwise comparisons. No samples were identified as ethnic outliers by use of identity by state clustering.

**Figure 2:**
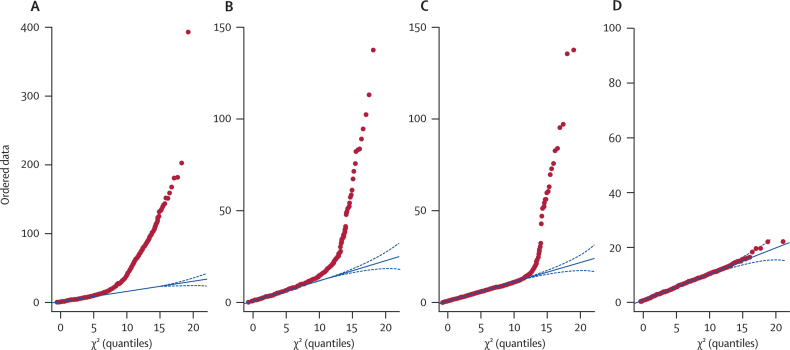
QQ (quartile–quartile) plots of different stages of quality control (A) Unfiltered allelic χ^2^ test of vCJD samples versus all UK samples (internal and Welcome Trust Case Control Consortium [WTCCC]) data. (B) Standard filtering allelic χ^2^ test of vCJD versus all UK samples (internal and WTCCC data). (C) Standard filtering with allelic χ^2^ test of vCJD versus only internal UK samples. (D) High-stringency filtering. With standard filtering, the inflation factor used for genomic control of confounding factors was estimated as 1·06 (1·01–1·09). Red dots=observed data. Blue lines=expected data. Broken blue lines=95% CI for expected data.

Genotype data quality analysis and filtering was done with PLINK. From 598 676 unfiltered SNPs, the following were excluded from further analysis by standard quality control: monomorphic SNPs or those not genotyped by EA-500K or WTCCC arrays (n=170 334); greater than 10% missing genotypes in vCJD (n=66 659) or WTCCC (n=9828); evidence of Hardy–Weinberg disequilibrium (exact test, p<0·001) in our UK samples (n=4873) or (exact test, p<1·0×10^−5^) in WTCCC samples (n=7673); minor allele frequency less than 0·01 in vCJD and WTCCC samples (n=57 853); allelic test for differences in our in-house UK samples versus WTCCC (p<0·001; n=1888). After this trimming, 410 287 SNPs remained for testing in 117 patients with vCJD versus 3083 UK controls (84 in-house UK controls and 2999 WTCCC samples). A more stringent filter applied additional thresholds of less than 3% missing data overall, and minor allele frequencies greater than 3% (n=288 908 SNPs remaining). These stringently filtered data were assessed for whether the skewed quartile–quartile (QQ) plots (figure 2) were caused by cryptic population stratification between the UK control and vCJD groups or alternatively by inaccurate SNP genotyping. The absence of a significantly skewed QQ plot in the stringently filtered data supports the hypothesis that SNPs were inaccurately called, probably on the EA-500K platform. Subsequently this was confirmed by concordance testing with the GoldenGate platform.

Candidate SNPs for further study were identified in stringently filtered dataset or after standard filtering if there was additional evidence of genotype accuracy, by identifying an association signal in nearby SNPs in strong linkage disequilibrium with the candidate SNP. As a further test to identify false-positive associations related to differential genotyping accuracy between cases and controls, we validated (>99% concordance) all genotypes shown in vCJD and in-house UK controls with an independent platform (MGB probe and quantitative PCR) before attempting replication in other categories of prion disease with the same technology (35 SNPs were tested in this way). To maximise coverage, a further 17 SNPs were chosen from the *PRNP* locus (by maximising pairwise *r*^2^ with HapMap build 35 using Haploview) and genotyped with GoldenGate technology. In total 52 SNPs were genotyped for association studies further to the discovery phase.

PLINK was used for association and permutation testing. The primary analysis was an allelic χ^2^ test with use of empirical p values if any cell count was less than 15. A secondary analysis implemented genotypic, dominant, and recessive models, with empirical significance if necessary, controlling for the four tests done. Imputation of codon 129 genotype was done by the PLINK proxy-impute command (multimarker tagging) with dense SNP data around *PRNP*, including rs1799990, generated in 344 in-house UK controls. A nominal genome-wide significance threshold of p<5×10^−7^ was used in the primary analysis in concordance with the WTCCC. Owing to the large number of SNPs that were tested, this threshold takes into account multiple-hypothesis testing. In the replication phase of the study, the small number of tested SNPs permits a less stringent threshold of p<0·001.

Population structure was analysed with IBS clustering (implemented through PLINK) and principle components analysis (implemented through the Eigenstrat package^19^). Genome-wide data that were filtered to high stringency were used to compare samples from patients with vCJD and UK controls with PLINK and Eigenstrat (no significant eigenvectors were detected with default procedures). A separate low-density study was done with GoldenGate technology for several reasons: to investigate genotype accuracy in the SNPs filtered out by the high-stringency filtering step; to provide evidence with regard to the population structure in the samples from Papua New Guinea, which was previously unknown; to provide evidence that concordant genotypes consistent with the healthy population frequencies could be obtained from the amplified degraded samples from patients with kuru. 17 additional SNPs were genotyped to provide dense coverage of the *PRNP* locus, which is a region that confers susceptibility to prion disease, with a high probability of novel susceptibility being discovered here. These 17 SNPs complemented an existing dataset of 25 SNPs from the earlier genome-wide phase of the study. SNPs were selected from standard stringency filtered data in the genome-wide phase of the study; all autosomes were equally represented, with a median intermarker distance of 1·3 Mb. 1523 individuals were genotyped for 1325 SNPs: 344 randomly selected, non-related, white blood donors from the UK provided by the ECCCs; 119 patients with vCJD; 485 patients with sCJD; 28 patients with iCJD; 143 patients with kuru; 122 elderly women who are resistant to kuru and were born before 1950; and 282 young individuals from the kuru region matched to the elderly women by village of residence (figure 1). These patients were a subset of those included in the replication studies. SNPs were filtered for association with vCJD by comparison with UK controls by best permuted p<0·001 from any of four genetic models (allelic, trend, genotypic, or recessive) with the GoldenGate platform at the St Bartholomew's Hospital Genome Centre. Genotyping quality was assessed by Hardy–Weinberg equilibrium (excluding those assessed by exact test p<0·001) and visual inspection of all genotype clusters with Beadstudio version 3.1. The overall genotype call rate was 99·7%, and concordance of duplicate samples was excellent (nine WGA2 degraded amplified kuru samples [concordance 99·7%] and 20 healthy control duplicates [concordance >99·9%]). This study confirmed that the skew in the QQ plots was caused by inaccurate genotyping of SNPs in our genome-wide study that were not adequately filtered by the low-stringency criteria. For Eigenstrat, ten eigenvectors were generated through default procedures and outlier detection (6 of 826 samples from Papua New Guinea were removed). No significant eigenvectors (p>0·01) were identified between patients with sCJD or iCJD and UK controls, or between patients with kuru, elderly women who are resistant to kuru, and healthy young Fore (five comparisons in total).

The Gene Expression Analysis Software (MAS 5.0) was used to analyse the raw image files from the quantitative scanning, which resulted in files that contained background corrected values for the probes. Significance analyses to compare prion versus mock-infected cells used a two-class unpaired test with a Benjamini–Hochberg (false discovery rate) p-value correction.

### Role of the funding source

The sponsors had no role in the study design, data collection, data analysis, data interpretation, or writing of the report. Simon Mead and John Collinge had full access to all the data in the study and final responsibility for the decision to submit for publication.

## Results

**Figure 3:**
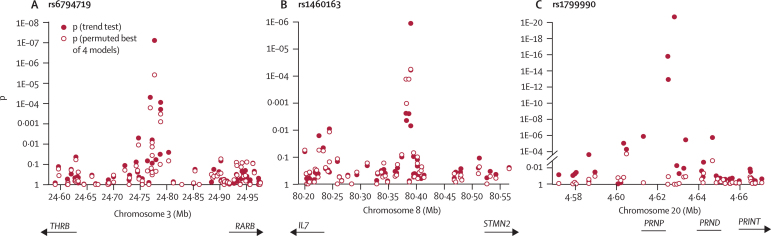
Physical location and p of allelic test and best of four genetic models (A) SNPs between *THRB* and *RARB*, including rs6794719. (B) SNPs upstream of *STMN2* including rs1460163. (C) SNPs at the *PRNP* locus, including rs1799990, rs6107516, and rs6116492, showing trend test (filled circles) and a test comparing vCJD with UK controls with codon 129 methionine homozygous genotypes (empty circles [imputed for WTCCC controls]).

After standard quality control for call rate and minor allele frequency, Hardy–Weinberg disequilibrium, and differences between control datasets, we analysed 410 287 SNPs in the primary analysis. QQ plots showed an excess of large allelic χ^2^ test statistics (figure 2) owing partly to the comparison between cases and controls among platforms and laboratories and was completely resolved by stringent filtering by call rate and minor allele frequency, leaving about 300K SNPs for association testing. Comparison of these stringently filtered data between vCJD and our own UK controls, ECACC controls, and WTCCC controls with Eigenstrat and GC methods did not provide evidence of significant population stratification; therefore, association statistics were not corrected. Similarly, we found no evidence of population stratification in comparisons of 1325 SNPs in replication cohorts (sCJD, iCJD, and control groups from the UK) or between patients with kuru, elderly women who were resistant to kuru, and healthy Fore from Papua New Guinea. In the stringently filtered data, two SNPs were significant at the genome-wide level (p<5×10^−7^) on the basis of allelic tests: rs6107516 in the intron of *PRNP* and rs6794719 in an intergenic region between *RARB* and *THRB*, which encodes thyroid hormone receptor beta (figure 3).

A block of linkage disequilibrium that was larger than ∼100 kb and included all of *PRNP* was shown by 25 SNPs (figure 3). We added 17 more SNPs from vCJD and UK controls, including *PRNP* codon 129 (rs1799990). Unsurprisingly, rs6107516, which is located in the intron of *PRNP* and is in moderately strong linkage disequilibrium with codon 129 (rs1799990, *r*^2^=0·6), was the top-ranked single SNP in the discovery phase. All patients with vCJD who have been genotyped to date are homozygous for rs1799990A (MM at *PRNP* codon 129). rs6107516 was most strongly associated in an allelic model (p=2·5×10^−17^ OR 38·5, 95% CI 9·6–155·2).

From the 25 SNPs in the discovery phase, codon 129 of *PRNP* was best tagged by a two SNP haplotype formed by rs6031692 and rs6107516 (*r*^2^=0·7, based on Hapmap build 35 data) with a haplotypic association of p=1×10^−24^. To test for more association at the locus, we conditioned for the association of codon 129 by imputing this genotype and including only methionine-homozygous UK controls from the WTCCC series. We thus identified evidence of additional genetic risk at this locus. rs6116492, which is downstream of *PRNP* and also in strong linkage disequilibrium with rs1799990, had a frequency of 0·06 in patients with vCJD and 0·017 in UK controls (allelic model p=8·2×10^−5^; 0·022 in 1544 UK controls with an imputed codon 129 methionine homozygous genotype; allelic model p=0·001, OR 2·63, 95% CI 1·43–4·82). rs6116492 is located in an intergenic region between *PRNP* and *PRND*, which encodes prion-like protein doppel. Genetic risk factors for sCJD have previously been identified upstream and downstream of *PRNP* but not for vCJD.^20^, ^21^ Because we cannot guarantee that the rs1799990 genotype has been imputed perfectly, we also compared cases of vCJD (n=119) with in-house UK controls genotyped at rs1799990 and rs6116492 (n=701); we again found a significant, independent association of rs6116492, both by haplotype test conditioned on codon 129 (PLINK, likelihood ratio test with one degree of freedom; p=0·037), or simply by excluding controls with methionine/valine or valine/valine encoded at codon 129 genotypes followed by an allelic test (119 patients with vCJD *vs* 294 in-house UK controls with the genotype that encodes methionine/methionine at codon 129; p=0·02). SNP-1368 (rs1029273C, 24 466 base pairs upstream of codon 129), which we and others have confirmed to be associated with sporadic CJD but not vCJD, also showed no evidence of association with vCJD independent of codon 129.^20^, ^21^

Because we tested the entire collection of samples from white British patients with vCJD (the majority of cases of vCJD), we then looked to closely related prion diseases to replicate independently and more broadly candidate SNPs with risk of prion disease. We tested for the association of rs6794719, rs6116492, and 33 other top-ranking SNP associations from the vCJD study in patients with iCJD who were exposed to prion disease through cadaver-derived growth hormone therapy versus UK controls (n=28); 506 patients with sCJD—a worldwide disease of uniform incidence that affects about 1–2 million people per year—versus UK controls. We also tested patients with kuru and healthy elderly women who were exposed to but survived the kuru epidemic from the Eastern Highlands Province of Papua New Guinea. In the groups from Papua New Guinea, we tested whether candidates for genetic risk of vCJD were associated with kuru incubation time (by comparison with a cohort of young-onset kuru [n=59] versus old-onset kuru [n=92]) or resistance to kuru, by comparison of elderly female survivors (n=125) with the young population (n=280–526). Homozygosity at codon 129 of *PRNP* was significantly associated with risk of iCJD (p=2·7×10^−4^), sCJD (p=2·3×10^−21^), and tests done in Papua New Guinea (Fisher's method p=2·2×10^−9^; Table 1, Table 2).

**Table 1:** Discovery tests of rs1799990 and four novel candidate SNPs in patients with vCJD and UK controls

	**Chromosome**	**Locus**	**Minor allele**	**Major allele**	**vCJD genotypes**	**UK control genotypes**	**Model**	**p (vCJD)**	**OR**
rs1799990	20	4628251	G	A	119/0/0	294/324/81	A	2·0×10^−27^	..
rs6107516	20	4625092	A	G	117/2/0	1960/1227/227	A	2·5×10^−17^	38·5 (9·6–155·2)
rs6116492	20	4646626	T	G	104/12/1	2979/104/0	A	8·2×10^−5^	3·71 (2·09–6·59)
rs1460163	8	80390003	A	G	7/25/86	31/657/2734	R	5·6×10^−5^	6·9 (3·0–16·0)
rs6794719	3	24777543	T	A	3/31/84	346/1465/1596	A	1·9×10^−7^	2·5 (1·7–3·7)

Genetic models: A=allelic; G=genotypic.

**Table 2:** rs1799990 and three novel candidate SNPs in samples from Papua New Guinea

	**Resistance to kuru despite exposure**				**Early-onset and late-onset kuru**				Combined p value*
	Elderly women exposed to kuru	Healthy young Fore	Model	p value	Young kuru (age <25 years)	Older kuru (age >25 years)	Model	p value (kuru incubation)	
rs1799990	16/86/23	112/287/163	G	0·001	16/18/25	9/71/12	G	9·1×10^−8^	2·2×10^−9^
rs6116492	80/37/2	393/151/18	A	0·848	47/11/0	57/20/0	A	0·44	..
rs1460163	30/77/16	140/144/40	R	2·5×10^−4^	23/29/5	26/42/23	A	0·017	5·7×10^−5^
rs6794719	5/47/63	26/118/136	A	0·12	4/25/27	7/33/47	A	0·687	..

Genetic models: A=allelic; G=genotypic; R=recessive.

* Fisher's method.

rs6794719A was associated with the risk of vCJD at a nominal genome-wide significance of p=1·9×10^−7^. The more frequent allele, rs6794719A, was also associated with disease risk in the small collection (n=28) of patients with iCJD (p=0·030; table 3) but not those with sCJD, kuru, or resistance to kuru.

**Table 3:** Replication tests of rs1799990 and four novel candidate SNPs in patients with iCJD, patients with sCJD, and UK controls

	**iCJD genotypes**	**UK control genotypes**	**Model**	**p (iCJD)**	**sCJD genotypes**	**Model**	**p (sCJD)**
rs1799990	4/13/11	294/324/81	A	2·7×10^−4^	307/98/101	G	2·3×10^−21^
rs6107516	9/12/7	1960/1227/227	R	0·002	320/100/55	G	3·6×10^−11^
rs6116492	28/0/0	2979/104/0	A	0·62	483/22/0	A	0·30
rs1460163	0/7/21	31/657/2734	A	0·66	7/93/396	A	0·83
rs6794719	1/8/19	346/1465/1596	A	0·03	61/207/207	A	0·08

Genetic models: A=allelic; G=genotypic; R=recessive.

From the 33 top-ranked SNPs that failed to achieve genome-wide significance in patients with vCJD, the strongest overall evidence of association in replication cohorts was for rs1460163 (combined p=6·3×10^−8^ by Fisher's method across orally acquired prion disease categories [combination of vCJD and Papua New Guinea tests]). rs1460163 was associated with age of kuru onset (p=0·017) and resistance to kuru (p=2·5×10^−4^), with the same highest-ranking risk allele for vCJD and kuru. rs1460163 is located in a large block of linkage disequilibrium that extends just 5′ to *STMN2* (figure 3). Other SNPs tested in the replication phase were either poorly genotyped in the discovery phase (concordance <99%), or showed no evidence of association in any prion disease category additional to vCJD (p>0·001; best from four risk models).

**Figure 4:**
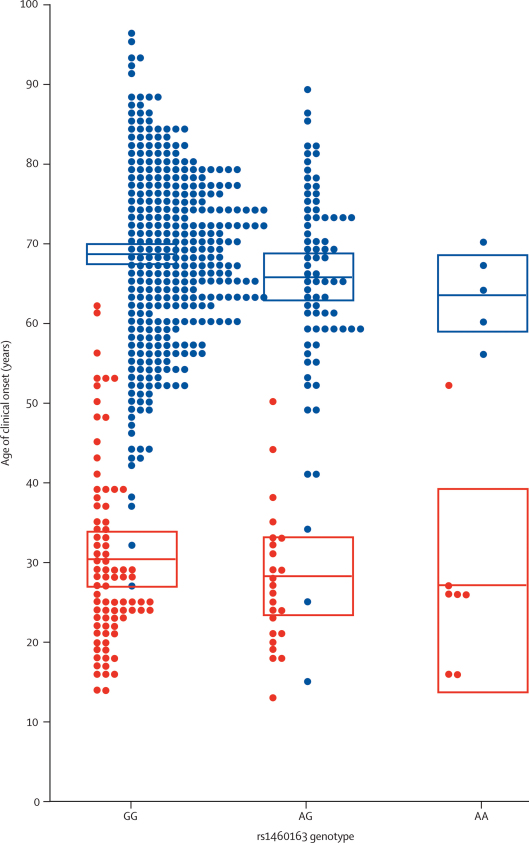
Age of clinical onset of vCJD (red) and sCJD (blue) patients against rs1460163 genotype Clinical onset was defined as the age of the first symptom that progressed into a neurological or neuropsychiatric condition due to prion disease. The central bars indicate mean age of onset; boxes indicate 95% CI of the mean.

We then analysed the clinical and molecular phenotype of UK prion disease for rs1460163, rs6116492, and rs6794719. In patients with sCJD, there was a significant modifying effect of the risk allele, with clinical onset 5 years earlier for those with risk genotype rs1460163AA compared with those with GG (linear regression of log-transformed age of onset against genotype p=0·02; figure 4). In patients with vCJD, the mean age of onset for genotype AA was 3 years earlier than for those with GG, but this was not statistically significant (p=0·26). By use of a linear regression model with disease type as a factor, the rs1460163AA allele was associated with age of onset in sCJD and vCJD (log-transformed p=0·01) and was also independent of rs1799990. No effect was seen on year of presentation, which in part will determine incubation time in vCJD, but this analysis is confounded by uncertainty in the time of exposure. No effect was seen on sCJD PrP^Sc^ strain type as defined by partial protease K digestion and western blot. rs6116492 and rs6794719 had no effect on prion disease phenotype.

**Figure 5:**
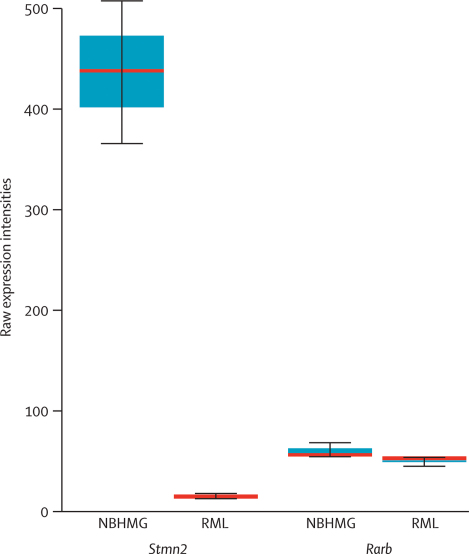
Boxplot of *Stmn2* and *Rarb* expression Expression of *Stmn2* and *Rarb* in mouse neuronal cells (GT-1) treated with homogenate of healthy brain (NBHMG) or Rocky Mountain Laboratory scrapie brain homogenate (RML). Median is shown as a thick red horizontal line, IQR by boxes, and largest and smallest observations by whiskers.

In a cellular model of mouse prion disease, the expression of *Stmn2* was profoundly altered by infection with prions. This difference was shown by comparison of the transcriptome of prion-infected and prion-uninfected cells in culture. Mouse hypothalamic neuronal (GT-1) cells that were infected with mouse brain homogenate (NBHMG) or RML-infected brain homogenate were analysed with the Affymetrix Mouse Expression Array 430_2.0. Comparison of the expression between NBHMG and RML showed that *Stmn2* is significantly (p=3·6×10^−18^) downregulated by a factor of about 30 and ranked tenth out of more than 21 537 genes that were represented by one or more transcripts on the array (figure 5). In this study, the expression of 543 of 21537 (2·5%) genes was altered, with a fold change of more than 2·83 (corrected Benjamini–Hochberg method). Neither *RARB* nor *STMN2* is significantly expressed in human blood cells, which obviates the analysis of the correlation of gene expression with genotypic risk in a large collection of samples.

## Discussion

We describe the first genome-wide study of genetic risk in a human prion disease and replication of a small number of top-ranking candidate SNPs. Further genetic studies of human prion disease, including more extensive replication studies, are warranted because our power was limited by the small size of the vCJD sample and an early generation platform was used. Owing to the rarity of the disease, all available samples were used; the use of amplified DNA in a proportion of cases might have also affected the quality of genotyping. For these reasons, we used highly stringently filtered data and verified genotypes from candidate SNPs with an in-house assay. The potential exists for a larger scale study in sCJD that capitalises on decades of surveillance for human prion diseases across Europe and the rest of the world; however, this disease is undoubtedly more heterogeneous than vCJD.

The potential overlap in pathogenesis between vCJD and the other prion disease categories used in the replication phases of the study must also be considered. The pathogenesis of vCJD contrasts with the replication cohorts in terms of prion strain (all groups), tissue distribution, and route of infection (for iCJD and sCJD). Furthermore, in the case of our large collection from Papua New Guinea, the linkage-disequilibrium relationship between candidate SNPs and a putative functional SNP is not known and can therefore differ from that in the UK. For these reasons, an absence of association in one or more replication categories does not preclude a genuine association in vCJD.

The precedent of codon 129 was important to inform the comparisons in the replication phase. All UK prion diseases have strong associations with homozygous genotypes; for vCJD, only the methionine homozygous genotype. However, the groups from Papua New Guinea are the most relevant in the replication phase because our only precedent of a major acquired human prion disease epidemic is kuru, which was historically transmitted by cannibalism and had a devastating effect on the Fore and neighbouring linguistic groups of the Eastern Highland region of Papua New Guinea.^5^ Kuru was extensively documented at its peak in the mid-20th century.^22^ We amplified DNA from this archive and continued surveillance of kuru in the Fore in the late 20th century to identify recent cases of kuru with long incubation times and elderly Fore women with long-term survival after exposure to high doses of prions. At *PRNP* codon 129, elderly Fore women survivors of the kuru epidemic showed a profound Hardy–Weinberg disequilibrium, with an excess of the prion disease-resistance genotype 129MV relative to both homozygous genotypes 129MM and 129VV. The patients with kuru show an age stratification of codon 129, with young patients being mostly genotype MM or VV and adult or elderly patients being mostly MV, consistent with a powerful effect of codon 129 MV in extending kuru incubation time.^5^, ^23^, ^24^ Our study thus confirms the strong association of *PRNP* codon 129 (rs1799990) across acquired and sporadic prion diseases as the outstanding genetic risk factor in human prion disease. Notably, the effect was detectable in a small sample, which should be encouraging for those contemplating studies of rare diseases with well characterised patients and a distinct pathogenesis.

The additional associations we report are not as strong or robust as those we confirm for *PRNP* codon 129 but each of these are beyond what would have been expected by chance when taking into account the problem with multiple testing. Although we cannot be certain that any of the three candidate SNPs we describe altered the expression of their nearest gene (*PRNP, STMN2*, or *RARB*), in each case these are excellent candidates for involvement in prion pathobiology. The risk conferred by rs6116492T could act through altered expression of *PRNP* owing to the crucial role for PrP in prion disease pathobiology; however, we have no direct evidence that a putative genetic risk conferred by rs1460163 or rs6794719 is manifest through their nearest genes (*STMN2* or *RARB*) because these SNPs have no linkage disequilibrium with coding regions. Regulatory regions often act on nearby genes but can also act over great distances or even on different chromosomes, implicating other genes.^25^

In the absence of further cohorts of orally acquired prion disease and taking into account the aforementioned caveats, we turn to functional evidence of a role for these candidate genes in prion disease. The expression of PrP in cultured neuronal and lymphoid cells is regulated by retinoic acid.^26^, ^27^, ^28^ Furthermore, the production of the disease-associated isoform of PrP (designated PrP^Sc^) in cultured mouse neuronal cells infected with mouse prions is increased by treatment with retinoic acid.^26^ Whether retinoic acid acts through the receptor encoded by *RARB* or another retinoic acid receptor for these biological activities is not known at present. In addition to *PRNP*, the strongest overall genetic evidence we found is for a SNP association upstream of *STMN2*. SCG10, the protein product of this gene, is a regulator of microtubule stability in neuronal cells, with potential implications for aggresome formation and modulation of prion neurotoxicity.^29^ We found that *Stmn2* is turned off by prion infection in mouse neuronal cells, in keeping with an early study,^30^ but different from a recent and rigorously conducted study.^31^ Whether prion infection or unknown experimental factors are responsible for this large effect is unclear; a role for SCG10 in prion infection has not been established and speculation about a mechanism in prion disease would be premature.

Our data lend considerable support to the hypothesis that genetic susceptibility in addition to *PRNP* codon 129 genotype has contributed significantly to the outbreak of vCJD to date. Whether these effects are on the incubation period rather than susceptibility, such that further waves of BSE-associated prion disease with longer incubation periods might occur in the years ahead and be associated with different genotypes at many risk loci, is unknown.^32^
